# Effects of Chemical and Mechanical Lysis on Microbial DNA Yield, Integrity, and Downstream Amplicon Sequencing of Rumen Bacteria and Protozoa

**DOI:** 10.3389/fmicb.2020.581227

**Published:** 2020-11-16

**Authors:** Zhi Yuan Ma, Xiu Min Zhang, Rong Wang, Min Wang, Ting Liu, Zhi Liang Tan

**Affiliations:** ^1^CAS Key Laboratory for Agro-Ecological Processes in Subtropical Region, National Engineering Laboratory for Pollution Control and Waste Utilization in Livestock and Poultry Production, Institute of Subtropical Agriculture, Chinese Academy of Sciences, Changsha, China; ^2^College of Resources and Environment, University of Chinese Academy of Sciences, Beijing, China; ^3^College of Animal Science and Technology, Gansu Agricultural University, Lanzhou, China

**Keywords:** chemical lysis, mechanical lysis, DNA extraction, rumen fluid, bacteria, protozoa

## Abstract

QIAamp Fast DNA Stool Mini Kit (QIAGEN, Valencia, CA, United States) and RBB + C (Yu and Morrison, 2004) methodologies are widely employed to extract microbial DNA from rumen samples and can exhibit different efficiencies of obtaining DNA yield, quality, and downstream amplicon sequence analysis. No study has conducted to investigate the contributions of chemical and mechanical lysis on DNA extraction, which included chemical lysis from QIAamp Fast DNA Stool Mini Kit (QIA) and RBB + C (YM), bead (BB), and sand beating (SB). Effects of chemical lysis and bead beating (BB) were investigated by conducting a 2 × 2 factorial-designed experiment with four methodologies, including QIA without (QIA−) and with BB (QIA + BB), and YM without (YM−) and with BB (YM + BB). Comparisons between bead and sand were conducted by comparing methodologies of YM + BB and YM + SB. Comparing with QIA, YM had lower (*P* ≤ 0.10) OD_260__/__280_ and diversity of ZOTUs and length polymorphism of protozoal amplicons but harvested greater (*P* ≤ 0.086) DNA from fibrolytic bacteria such as *Ruminococcaceae* lineages. Including BB increased (*P* = 0.001) total DNA yield without affecting (*P* ≥ 0.55) OD_260__/__280_ and richness of bacterial ZOTUs but decreased (*P* ≤ 0.08) richness of both ZOTUs and length polymorphism of protozoal amplicon. Bead beating and SB showed no difference (*P* ≥ 0.19) in DNA yield and quality and bacterial and protozoal community. In summary, chemical lysis provided by RBB + C and QIAamp Fast DNA Stool Mini Kit should be better to extract DNA for analyzing bacterial and protozoal community, respectively. Sand can be an alternative beater for DNA extraction, and mechanical lysis is not recommended for protozoal community analysis.

## Introduction

The quality (yield, purity, and integrity) of microbial DNA extracted from digesta samples is crucial for downstream analysis of amplicon sequencing (Henderson et al., 2013; Vaidya et al., 2018). Both QIAamp Fast DNA Stool Mini Kit (QIAGEN, Valencia, CA, United States) and RBB + C (Yu and Morrison, 2004) methodologies are widely used to extract microbial DNA from rumen samples. However, the RBB + C recovers greater microbial DNA yield from rumen fluid samples with 5-fold increase when compared to QIAamp Fast DNA Stool Mini Kit, implicating a more suitable methodology to extract microbial DNA from rumen fluid. Furthermore, downstream microbial community analysis showed distinctions between these two methodologies based on DGGE (Yu and Morrison, 2004) and amplicon sequencing (Henderson et al., 2013; Vaidya et al., 2018). Comparing with QIAamp Fast DNA Stool Mini Kit, RBB + C methodology not only has a different chemical lysis but also includes bead beating. Contributions of chemical lysis and bead beating have not been investigated in these two DNA extraction methodologies.

Mechanical lysis is another factor that influences efficiency of DNA extraction. Evidences show that including a beader beating step improves DNA yield (de Boer et al., 2010; Guo and Zhang, 2013; Pollock et al., 2018), bacterial diversity (Guo and Zhang, 2013), gram-positive bacteria (Salonen et al., 2010), spores (Salonen et al., 2010), and fungi (Fiedorova et al., 2019). Sand is raw material of bead and has a similar chemical composition of silicon dioxide as bead. Sand is irregularly shaped and may disrupt cell walls more efficiently than the evenly shaped round bead. Furthermore, sand is cheaper and more available than bead; replacing bead with sand may decrease the costs for DNA extraction. Including sand sufficiently extracts DNA from human feces for detecting pathogens (Karasartova et al., 2018). However, no study has been performed to evaluate the effect of sand beater on the efficiency of DNA extraction from rumen microorganisms.

The first objective of this study was to compare the effects of chemical lysis provided by RBB + C and QIAamp Fast DNA Stool Mini Kit and bead beating on microbial DNA quality and downstream amplicon analysis. The second objective is to compare bead and sand beating on microbial DNA quality and downstream amplicon analysis.

## Materials and Methods

All animal procedures followed our institutional guidelines for the care and use of animals and were approved by the Animal Care Committee (Approval number ISA-W-201609), Institute of Subtropical Agriculture, Chinese Academy of Sciences, Changsha, China.

### Experimental Design, Rumen Sampling, and DNA Extraction

**FIGURE 1:**
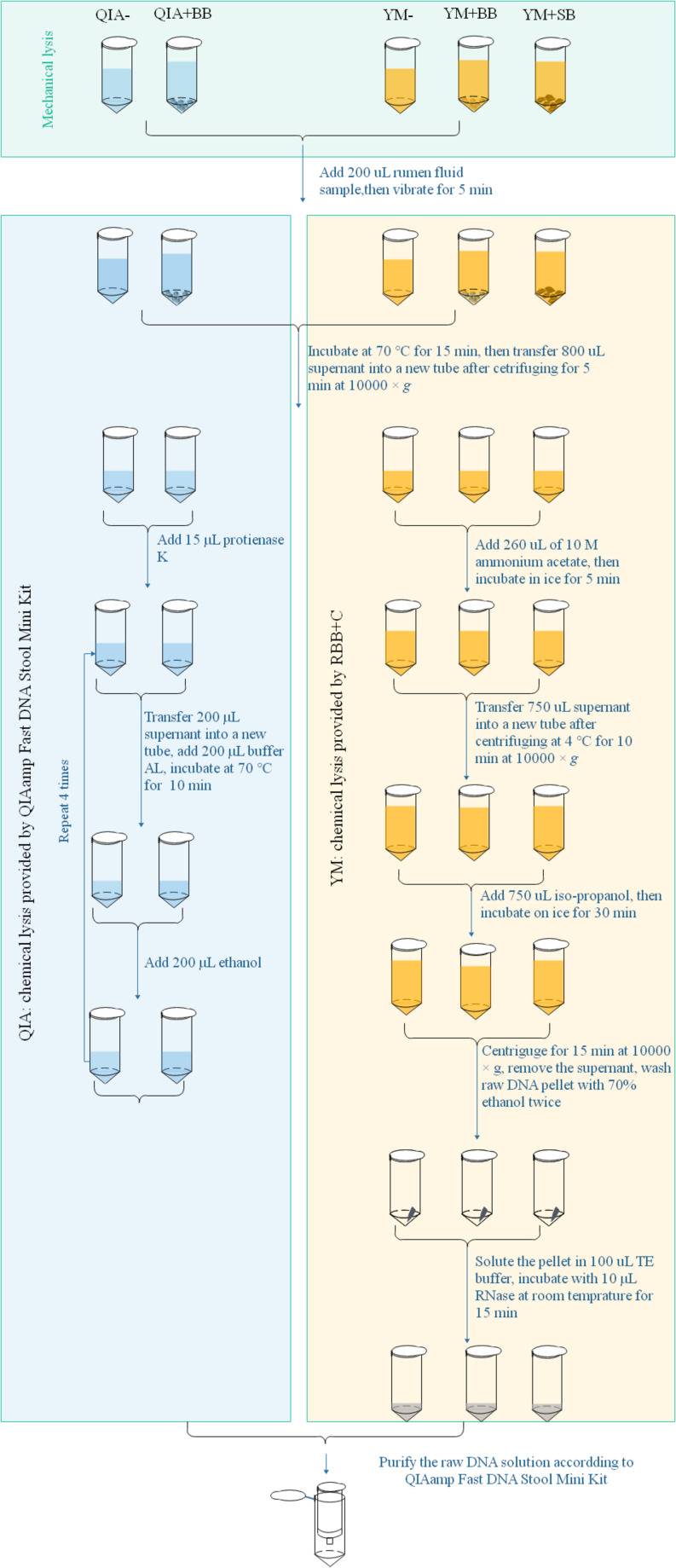
Scheme for DNA extraction procedures of five experimental methodologies. QIA, the chemical lysis provided by QIAamp Fast DNA Stool Mini Kit methodology; YM, the chemical lysis provided by RBB + C (Yu and Morrison, 2004); BB, bead beating; SB, sand beating; QIA–, QIA without mechanical lysis; QIA + BB, QIA with BB, YM–, YM without ML; YM + BB, QIA with BB; YM + SB, QIA with SB.

Microbial DNA extraction briefly had three major steps from protocol of QIAamp Fast DNA Stool Mini Kit and RBB + C methodologies. Mechanical lysis (ML) was defined as pretreated processes with mechanical breakdown such as a violent vibration, while chemical lysis (CL) was defined as the chemical environment provided by processes assorted to their lysis buffers after ML to acquire raw DNA (Figure 1). The final purification was the processes to acquire good-quality DNA using silica matrix columns (Figure 1). QIAamp Fast DNA Stool Mini Kit methodology exhibited a different chemical lysis (QIA) in comparison with that of RBB + C methodology (YM) and did not contain a step of mechanical lysis. Mechanical lysis is a beating-based step and included bead (BB, 100–200 μm mesh, Omega Bio-Tek, Norcross, GA, United States) or sand (SB, 300–800 μm mesh, Sinopharm Chemical Reagent Co., Ltd., China) beating.

The procedure of five experimental methodologies included QIA without (QIA−) and with BB (QIA + BB), and YM without (YM−) and with BB (YM + BB) or SB (YM + SB), which were briefly described in Figure 1. Briefly speaking, for methodologies without beater beating including QIA− and YM−, samples were vibrated with 1 mL of designated lysis buffer. For methodologies with beater beating including QIA + BB, YM + BB, and YM + SB, 200 mg bead or sand was further added before vibrating. It needs to be noted that the original procedure of RBB + C methodology had a repeated lysis step to debris, which was discarded to ensure that the only difference between QIA− and YM− methodology came from two lysis buffers. Effects of CL and BB were investigated by conducting a 2 × 2 factorial-designed experiment with four methodologies, including QIA without (QIA−) and with BB (QIA + BB), and YM without (YM−) and with BB (YM + BB). Comparisons between bead and sand beading were conducted by comparing methodologies of YM + BB and YM + SB. Both two experiments share the YM + BB methodology.

Three rumen-cannulated goats aged 2, 5, and 6 years were used in this study. All goats were fed individually, and the diet was rice straw and concentrate (554 g corn grain, 198 g wheat bran, 185 g soybean meal, 30 g soybean oil, 12 g calcium carbonate, 11 g sodium chloride, and 10 g premix with vitamins and microelements per kg of DM) separately. The rumen contents were collected through the cannula 2 h after commencement of morning feeding. About 200 mL of rumen fluid was prepared for each animal by filtering the collected rumen contents through four layers of sterile cheesecloth into a prewarmed insulated bottle. Fresh samples were transferred to our laboratory within 10 min after detachment from rumen for microbial DNA extraction.

Microbial DNA extraction was conducted in triplicate by using 200 μL of rumen fluid sample of each goat according to the protocol of each methodology. All methodologies were vibrated by a vibrator (CEBO-48, Chebo BioTech Co., Shanghai, China) at a frequency of 50 Hz for 3 min, and raw DNA was purified through spin columns from QIAamp DNA Stool Mini Kit. The concentration and purity of DNA were measured using a spectrophotometer (ND-2000; NanoDrop Technologies, Wilmington, DE, United States). Two-microliter DNA samples were visualized by 1% agarose gel electrophoresis after pooling according to animals and methodologies.

### Amplicon Sequencing and Bioinformatic Analysis

The V4 region amplicons of rrs genes were achieved by using primes of GTGYCAGCMGCCGCGGTAA (forward, 5′→3′) and GGACTACNVGGGTWTCTAAT (reverse, 5′→3′) for bacteria (Walters et al., 2015), and CGCGGTAATTCCAGCTCCA (forward, 5′→3′), TTGGYRAATGCTTTCGC (reverse, 5′→3′) for protozoa (Hugerth et al., 2014). Amplicon PCR procedure was performed by following previously described methods by Ma et al. (2018) and sequenced using the Illumina MiSeq platform by Allwegene Tech., Beijing, China. The sequences without adaptors were then demultiplexed according to their inserted barcodes for bioinformatic analysis.

The barcodes and primers were stripped by using vsearch (Rognes et al., 2016). The stripped fastq pairs were merged by usearch v11 (Edgar, 2010). Quality controls of merged sequences were performed according to a vsearch pipeline^1^. Length filtered sequences were achieved by setting as -*fastq_minlen 248* -*fastq_maxlen 255* for bacteria, and -*fastq_minlen 50* -*fastq_maxlen 500* for protozoa. The chimera, singletons, and doubletons were removed after dereplicating of sequences. The ZOTUs were clustered by using usearch v11 (Edgar, 2010). The ZOTU table with amplicon counts of each ZOTU was created by mapping length-filtered sequences to representative ZOTU sequences.

The downstream analysis of taxonomical annotation and beta diversity based on Bray–Curtis dissimilar matrix (Bray and Curtis, 1957) were performed with Mothur v 1.41.1 (Schloss et al., 2009) according to the instruction of MiSeq SOP^2^. The reference of Silva.nr.132 (Quast et al., 2013) was used for bacteria and protozoa putative taxonomy annotation. The alpha diversity of ZOTUs was estimated by using ACE and Pielou as richness and evenness, respectively. The principle coordination analysis plots and genera relative abundance was visualized by ggplots2^3^.

### Statistical Analyses

All data was analyzed using the *lm* model by R v3.6.3^4^ except the relative abundance of microbial genera. The analysis model of first experiment was expressed as follows:

Y=i⁢k⁢mjμ+A+iC+kB+mC×kB+meji⁢k⁢m

where *Y*_*ikmj*_ is the response; μ is the general mean; *A*_*i*_ is the effect of animals (*i* = 3); *C*_*k*_ is the effect of chemical lysis (*k* = 2); *B*_*m*_ is the fixed effect of bead beater (*m* = 2); and *e*_*ikmj*_ is the random error term. When significant interactions occurred, a pair-wise comparison was conducted to determine differences among the four methodologies. Differences of *P* ≤ 0.05 were considered significant and 0.05 < *P* ≤ 0.1 were accepted as tendencies.

The analysis model of second experiment was expressed as follows:

Y=i⁢kjμ+A+iM+keji⁢k

where *Y*_*ikmj*_ is the response; μ is the general mean; *A*_*i*_ is the effect of animals (*i* = 3); *C*_*k*_ is the effect of mechanical lysis (*k* = 2); and *e*_*ikj*_ is the random error term.

As relative abundance of microbial taxa commonly does not fit a normal distribution, we used a permutational multivariate analysis of variance (PMAV) method (Fisher, 1925) for linear models described above. The PMAV was performed by *lmperm* v 2.1.0^5^, and permutation time was determined by the method of Anscombe (1953) by setting *perm* = *“Prob*.” The Bray–Curtis dissimilarity matrix among sources of variation was parted according to the algorithm proposed by Anderson (2001) and was performed by *vegan* v2.5 (Dixon, 2003) with 9999 permutation. All *P*-values were adjusted according to the method of Benjamini and Hochberg (1995) by *p.adjust()* function of R v3.6.3 (see text footnote 4).

## Results and Discussion

### DNA Yield

**FIGURE 2:**
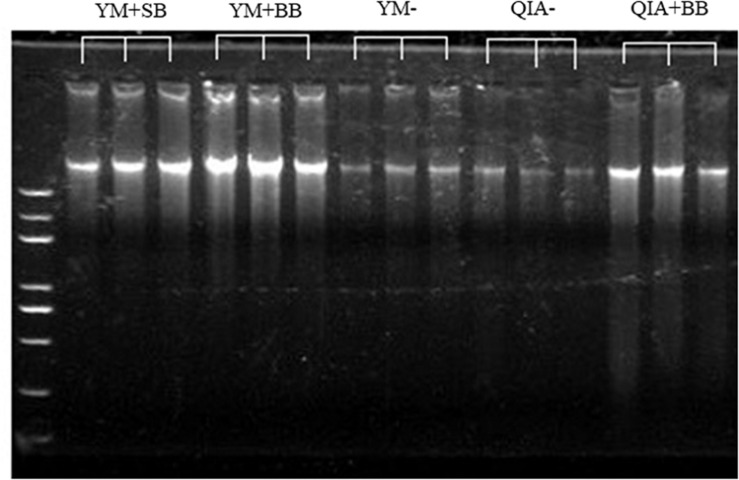
The agarose-gel electrophoresis graph of extracted DNA of five experimental methodologies. QIA, the chemical lysis provided by QIAamp Fast DNA Stool Mini Kit methodology; YM, the chemical lysis provided by RBB + C (Yu and Morrison, 2004); BB, bead beating; SB, sand beating; QIA–, QIA without mechanical lysis; QIA + BB, QIA with BB; YM–, YM without ML; YM + BB, QIA with BB; YM + SB, QIA with SB.

It has been widely reported that RBB + C extracts more microbial DNA from rumen fluid samples than QIAamp Fast DNA Stool Mini Kit (Yu and Morrison, 2004; Henderson et al., 2013). Such result can be caused by BB in RBB + C methodology, as the original QIAamp Fast DNA Stool Mini Kit methodology does not contain BB. Our results indicated that CL did not exhibit different DNA yields (*P* = 0.14, Table 1), indicating a similar efficiency to harvest DNA by QIA and YM. However, both agarose-gel electrophoresis image (Figure 2) and OD_260_ measurement indicated that BB greatly enhanced (*P* = 0.001; Table 1) DNA yield. Previous studies also indicate that BB effectively increases DNA yield in fecal samples (de Boer et al., 2010; Karasartova et al., 2018). Furthermore, the lack of interaction between CL and BB was another important finding of this study (*P* ≥ 0.48, Table 1), which indicates that the improved DNA yield caused by BB is independently on CL.

**TABLE 1:** Effect of chemical and mechanical lysis on DNA yield, quality, alpha diversity of bacterial and protozoal ZOTU, and length polymorphism of protozoal amplicons (*n* = 3).

**Item**	**Methodology^1^**					**Experiment 1^2^**				**Experiment 2^3^**	
			
						**SEM**	***P*-value**			**SEM**	***P*-value**
					
	**QIA−**	**QIA + BB**	**YM−**	**YM + BB**	**YM + SB**		**CL**	**BB**	**CL × BB^3^**		

**Yield and quality of microbial DNA**											
Yield, μg/mL rumen fluid	36.2	53.0	39.1	62.7	60.7	2.25	0.14	0.001	0.86	3.50	0.72
OD_260__/__280_	2.33	2.22	1.75	1.82	1.81	0.021	<0.001	0.55	0.48	0.02	0.75
OD_260__/__230_	1.55	1.59	1.60	1.59	1.60	0.034	0.57	0.65	0.48	0.044	0.89
Amplicon read numbers											
Bacteria	36691	30586	33040	31906	35285	827.4	0.70	0.25	0.42	794.7	0.42
Protozoa	81624	26971	26581	19750	24280	11206.0	0.097	0.11	0.18	3520.3	0.46
Bacterial ZOTU											
ACE	1255	1185	1164	1124	1163	86.2	0.78	0.95	0.48	168.7	0.83
Pielou, × 10^–2^	72.1	75.3	74.3	74.8	73.6	1.12	0.63	0.15	0.30	0.92	0.44
Protozoal ZOTU											
ACE	1516	772	682	102	165	163.5	0.05	0.08	0.81	30.5	0.38
Pielou, × 10^–2^	52.8	46.6	57.1	60.6	46.8	2.15	0.017	0.68	0.15	7.16	0.41

**Length polymorphism of protozoal amplicons**											

ACE	340.1	287.9	146.5	68.8	127.5	28.15	0.10	0.002	0.76	44.35	0.45
Pielou, × 10^–2^	85.3^*A*^	52.8^*B*^	41.2^*B*^	38.1^*B*^	32.1	1.29	0.003	0.002	0.003	3.96	0.40
Bacteroidetes/Firmicutes	3.80	4.21	2.83	2.99	3.27	0.517	0.08	0.60	0.81	0.665	0.78

*CL, chemical lysis; ML, mechanical lysis; QIA, the chemical lysis provided by QIAamp Fast DNA Stool Mini Kit methodology; YM, the chemical lysis provided by RBB + C (Yu and Morrison, 2004); BB, bead beating; SB, sand beating. ^1^The five experimental methodologies includes QIA without ML (QIA−), QIA with BB (QIA + BB), YM without ML (YM−), QIA with BB (YM + BB), and QIA with SB (YM + SB). ^2^Experiment 1 is a 2 × 2 factorial analysis and includes four methodologies, such as QIA−. QIA + BB, YM−, and YM + BB. ^3^Experiment 2 compares YM + BB and YM + SB. ^*AB*^:Different superscript letters show significant difference between levels of one factor at each level of another factor when interaction is observed between CL and BB.*

Bead size can be another factor that influences efficiency of DNA extraction. Rantakokko-Jalava and Jalava (2002) reported that the smaller bead beating has greater DNA extracted from mock bacterial samples, as smaller beads provide greater relative surface and hence interact with large numbers of bacteria. In our study, although the mesh size of sand (300–800 μm) was three times greater than that of bead (100–200 μm), the DNA yield was not statistically different between BB and SB (*P* = 0.72, Table 1). It seems that both BB and SB have similar efficiency to harvest DNA yield. We speculate that the irregular shape of sand may compensate its disadvantage in mesh size.

### DNA Quality

The OD_260__/__280_ value of microbial DNA solution is an important indicator of DNA quality and can be increased by RNA remains or the hyperchromic effect of degraded microbial DNA (Felsenfeld and Sandeen, 1962; Desjardins and Conklin, 2010). Fiedorova et al. (2019) reported that RBB + C methodology produces a lower OD_260__/__280_ value than QIAamp Fast DNA Stool Mini Kit methodology (2.06 vs. 2.20). We suspect that such better DNA quality extracted by RBB + C methodology can be caused by YM, which has not been reported before. In our study, YM had lower OD_260__/__280_ closer to the ideal value of 1.8 than QIA (*P* < 0.001, Table 1). Such observation was further upheld by agarose gel electrophoresis image, especially that YM + BB methodology exhibited a lower shearing effect than QIA + BB methodology (Figure 2). It seems that chemical lysis of RBB + C methodology tends to obtain a better DNA quality of rumen samples. Although beater beating can increase the efficiency of DNA extraction, it may cause server shearing of DNA (Yu and Morrison, 2004), which can be revealed by a poor DNA integrity with decreased OD_260__/__280_ values (Desjardins and Conklin, 2010) and a diffused DNA band in agarose gel electrophoresis images (Yu and Morrison, 2004). However, our study showed that BB and SB had similar DNA integrity (Figure 2) and OD_260__/__280_ value (Table 1), indicating unchanged fragmenting DNA proportion by ML. Both QIAamp Fast DNA Stool Mini Kit and RBB + C methodologies have an identical final purification step, which is supposed to have similar ability to remove contaminants in the final DNA solution. In consistency with this theory, the OD_260__/__230_ value was not affected (*P* ≥ 0.48) by CL, BB, or SB in our study (Table 1). We speculate that YM helps to obtain a better quality of DNA extracted from rumen fluid samples, while proper use of ML may not affect the DNA quality.

### DNA Amplicon Sequence Analysis of Bacteria

**FIGURE 3:**
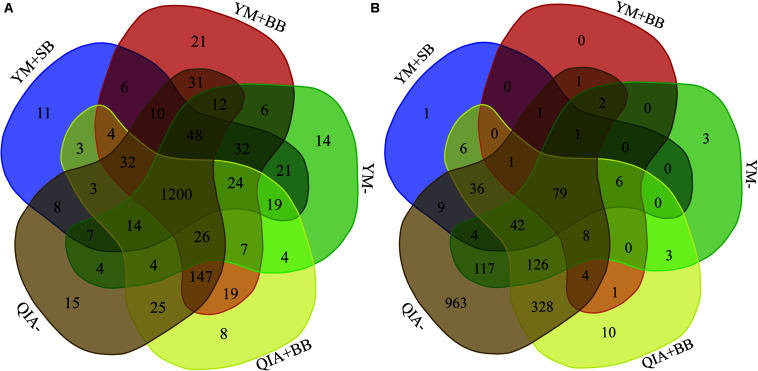
Venn diagram of bacterial **(A)** and protozoal **(B)** ZOTUs of five experimental methodologies. QIA, the chemical lysis provided by QIAamp Fast DNA Stool Mini Kit methodology; YM, the chemical lysis provided by RBB + C (Yu and Morrison, 2004); BB, bead beating; SB, sand beating; QIA–, QIA without mechanical lysis; QIA + BB, QIA with BB; YM–, YM without ML; YM + BB, QIA with BB; YM + SB, QIA with SB.

**FIGURE 4:**
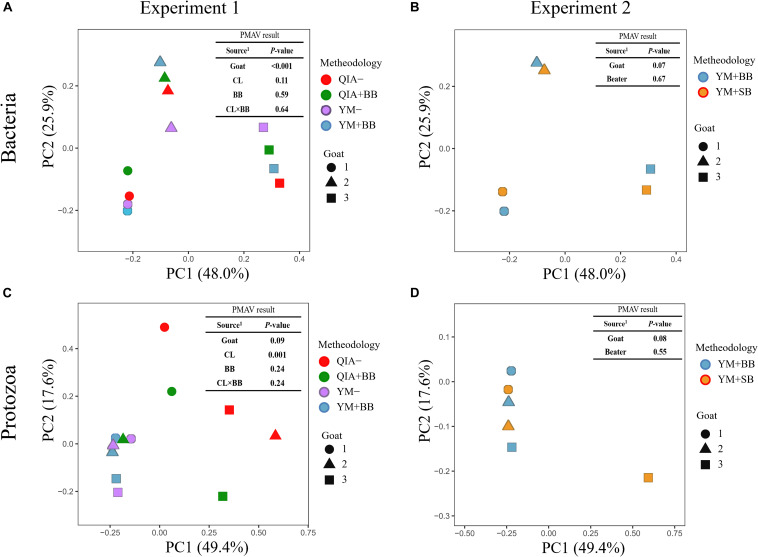
Principal coordinate analysis (PCoA) plots for the community of bacteria **(A,B)** and protozoa **(C,D)** based on the Bray–Curtis dissimilarity matrix. QIA, the chemical lysis provided by QIAamp Fast DNA Stool Mini Kit methodology; YM, the chemical lysis provided by RBB + C (Yu and Morrison, 2004); BB, bead beating; SB, sand beating; QIA–, QIA without mechanical lysis; QIA + BB, QIA with BB; YM–, YM without ML; YM + BB, QIA with BB; YM + SB, QIA with SB; PMAV, permutational multivariate analysis of variance with 999 permutations. Experiment 1 is a 2 × 2 factorial analysis and includes four methodologies, such as QIA–, QIA + BB, YM–, and YM + BB **(A,C)**. Experiment 2 compares YM + BB and YM + SB **(B,D)**.

It has been reported that RBB + C and QIAamp Fast DNA Stool Mini Kit methodologies have similar overall bacteria community (Fiedorova et al., 2019). In this study, we collected the rumen fluid samples from three goats with ages being 2, 5, and 6 years old, which were supposed to have different microbial communities. The five employed DNA extraction methodologies generated a total of 1785 bacteria ZOTUs and shared a total of 1200 bacterial ZOTUs (Figure 3A). The PCoA cluster analysis clearly indicated that animals (*P* < 0.001), other than CL and BB, were clearly separated (Figure 4A), indicating that the bacterial community was different among three goats and can be compared by using four emploryed DNA extraction methodologies in the first experiment.

**FIGURE 5:**
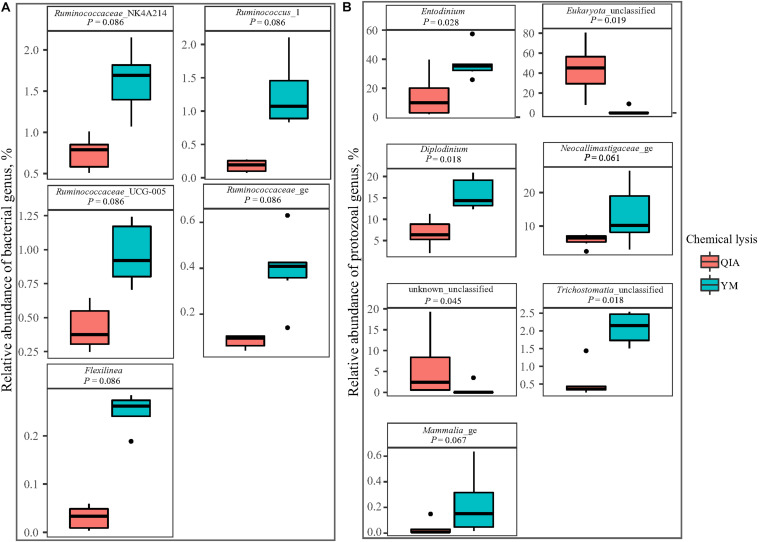
Boxplot of influenced (*P* ≤ 0.10) relative abundance of bacterial **(A)** and protozoal **(B)** genera by chemical lysis. QIA, the chemical lysis provided by QIAamp Fast DNA Stool Mini Kit methodology; YM, the chemical lysis provided by RBB + C (Yu and Morrison, 2004).

Fibrolytic bacteria have the ability to infiltrate the fiber surface layer, therefore tightly attaching to it (McAllister et al., 1994) and making it more difficult to be lysed by microbial DNA extraction methodologies (Whitehouse et al., 1994). We speculate that sodium dodecyl sulfate (SDS), an ionic surfactant which can dissolve non-fiber contents in measuring NDF, may also help to release fibrolytic bacteria. In consistency with this speculation, YM tended to have greater (*P* ≤ 0.086) relative abundance of *Ruminococcaceae* lineage of *Ruminococcus*_1, *Ruminococcaceae*_NK4A214, *Ruminococcaceae*_UCG-005, and *Ruminococcaceae*_ge, in comparison with QIA (Figure 5 and Supplementary Table S1). Although CL did not affect (*P* = 0.11, Figure 4A) the overall bacterial community, YM increased (*P* ≤ 0.086, Figure 5 and Supplementary Table S1) some fibrolytic bacterial genera with relative abundance being ranged from 0.33 to 0.78%.

The cell wall of gram-positive bacteria is more difficult to be lysed than that of gram-negative bacteria (Pollock et al., 2018). Including mechanical lysis, such as beater beating, has been reported to enhance hard lysis cells such as *Firmicutes* in fecal samples (Salonen et al., 2010). However, in our study, BB did not alter the overall bacteria community (Figure 4A) and relative abundances of major bacterial genera (>0.1%, Supplementary Table S1), as well as the ratio of major gram-negative bacteria to major gram-positive bacteria (i.e., Bacteroidetes/Firmicutes) (*P* = 0.60, Table 1). Furthermore, both BB and SB also exhibited a similar overall bacteria community (Figure 4B). It seems that proper use of ML has similar efficiency in lysis of different bacterial groups, leading to an unchanged relative abundance of hard lysis bacteria in rumen fluid samples.

### DNA Amplicon Sequence Analysis of Protozoa

The employed five DNA extraction methodologies generated a total of 1785 protozoal ZOTUs (Figure 3B), indicating that protozoa had almost similar ZOTUs with bacteria. Such result is contrary to the theory that diversity of protozoa is much lower than bacteria (Mcsweeney and Mackie, 2012). A total of 79 protozoal ZOTUs was shared by five DNA extraction methodologies and contributed to 89.4% of total reads of protozoa. Although the number of protozoal ZOTUs was unexpected large, protozoal ZOTUs representing the majority reads were reasonable.

Unlike the bacterial community, the PCoA cluster analysis indicated that both CL and animals separated the protozoal community (*P* ≤ 0.09, Figure 4A). The QIA increased the unclassified protozoa than YM (about 24-fold; *P* ≤ 0.045, Figure 5 and Supplementary Table S2). As only 29 *ciliates* and seven *Flagellate* genomes are known, higher unclassified protozoal genera generated by QIA may be caused by lacking protozoal gene contents in the Silva.nr.132 (Quast et al., 2013) database. Length polymorphisms of marker genes of eukaryotes such as protozoa are diverse (Hugerth et al., 2014). The QIA tended to generate more protozoal amplicons and had higher richness in amplicon length polymorphism than YM (*P* ≤ 0.1, Table 1). We speculate that the QIA can harvest DNA from greater diverse protozoa cells than YM.

Mechanical lysis is not recommended in extracting high-molecular-weight DNA (Rosewarne et al., 2011). The genome of protozoa is large, which can be vulnerated to mechanical lysis such as beater beating. For example, the genome of *Entodinium caudatum*, the most abundant protozoa in rumen, is 107 Mb^6^, which is about 30 times as rumen bacteria. In our study, neither the BB nor the SB influenced the overall protozoal community (*P* ≥ 0.24, Figures 4C,D) or annotation results (*P* ≥ 0.17, Supplementary Table S2). However, BB decreased (*P* ≤ 0.08) the richness of protozoal ZOTUs and length polymorphism of protozoal amplicons (Table 1). As limited protozoal genomes are included in the reference, only 6.9% protozoal ZOTUs were annotated at the genus level. The diversity of protozoal ZOTUs and length polymorphism can better indicate protozoal diversity than annotation results. Although ML did not influence overall protozoal community, it greatly decreased the richness of protozoa.

In summary, the QIA harvests DNA from higher diverse protozoa cells in comparison with YM. Although the YM has a similar bacterial community as QIA, it can help to harvest more DNA from fibrolytic bacteria such as *Ruminococcaceae* lineages. Proper use of ML can increase total DNA yield without affecting DNA quality and bacterial community but decreases protozoal amplicon diversity. Sand can be an alternative beater for DNA extraction, as both BB and SB have similar total DNA yield and quality and bacterial and protozoal community. Our study highlights that DNA extraction methodology should be slightly different for analyzing the bacterial and protozoal community.

## Data Availability Statement

The datasets presented in this study can be found in online repositories. The names of the repository/repositories and accession number(s) can be found below https://www.ncbi.nlm.nih.gov/, PRJNA644440.

## Ethics Statement

The animal study was reviewed and approved by the Animal Care Committee of Institute of Subtropical Agriculture, CAS (Approval number ISA-W-201609).

## Author Contributions

ZM, XZ, and MW conceived this study. ZM, XZ, and RW conducted this experiment. ZM and XZ carried out the statistical analysis. ZM and MW wrote the manuscript. TL and ZT revised the manuscript. All authors contributed to the article and approved the submitted version.

## Conflict of Interest

The authors declare that the research was conducted in the absence of any commercial or financial relationships that could be construed as a potential conflict of interest.

## Footnotes

1 https://github.com/torognes/vsearch/wiki/VSEARCH-pipeline

2 https://mothur.org/wiki/MiSeq_SOP

3 https://github.com/tidyverse/ggplot2

4 https://www.r-project.org/

5 https://github.com/mtorchiano/lmPerm

6 https://www.ncbi.nlm.nih.gov/genome/?term=Entodinium

## References

[B1] Anderson M. J. (2001). A new method for non-parametric multivariate analysis of variance. *Austr. Ecol.* 26 32–46. 10.1111/j.2517-6161.1953.tb00121.x

[B2] Anscombe F. J. (1953). Sequential estimation. *J. R. Statist. Soc.* 15 1–29.

[B3] Benjamini Y. Hochberg Y. (1995). Controlling the false discovery rate: a practical and powerful approach to multiple testing. *J. R. Statist. Soc.* 57 289–300. 10.1111/j.2517-6161.1995.tb02031.x

[B4] Bray J. R. Curtis J. T. (1957). An ordination of the upland forest communities of southern Wisconsin. *Ecol. Monogr.* 27 325–349. 10.2307/1942268

[B5] de Boer R. Peters R. Gierveld S. Schuurman T. Kooistra-Smid M. Savelkoul P. (2010). Improved detection of microbial DNA after bead-beating before DNA isolation. *J. Microbiol. Methods* 80 209–211. 10.1016/j.mimet.2009.11.009 19995580

[B6] Desjardins P. Conklin D. (2010). NanoDrop microvolume quantitation of nucleic acids. *J. Vis. Exp.* 2010:2565. 10.3791/2565 21189466 PMC3346308

[B7] Dixon P. (2003). VEGAN, a package of R functions for community ecology. *J. Veg. Sci.* 14 927–930. 10.1111/j.1654-1103.2003.tb02228.x

[B8] Edgar R. C. (2010). Search and clustering orders of magnitude faster than BLAST. *Bioinformatics* 26 2460–2461. 10.1093/bioinformatics/btq461 20709691

[B9] Felsenfeld G. Sandeen G. (1962). The dispersion of the hyperchromic effect in thermally induced transitions of nucleic acids. *J. Mol. Biol.* 5 587–610. 10.1016/s0022-2836(62)80088-6 13962882

[B10] Fiedorova K. Radvansky M. Nemcova E. Grombirikova H. Bosak J. Cernochova M. (2019). The impact of DNA extraction methods on stool bacterial and fungal microbiota community recovery. *Front. Microbiol.* 10:821. 10.3389/fmicb.2019.00821 31057522 PMC6479168

[B11] Fisher R. A. (1925). *Statistical Methods for Research Workers.* New York, NY: Hafner.

[B12] Guo F. Zhang T. (2013). Biases during DNA extraction of activated sludge samples revealed by high throughput sequencing. *Appl. Microbiol. Biotechnol.* 97 4607–4616. 10.1007/s00253-012-4244-4 22760785 PMC3647099

[B13] Henderson G. Cox F. Kittelmann S. Miri V. H. Zethof M. Noel S. J. (2013). Effect of DNA extraction methods and sampling techniques on the apparent structure of cow and sheep rumen microbial communities. *PLoS One* 8:e74787. 10.1371/journal.pone.0074787 24040342 PMC3770609

[B14] Hugerth L. W. Muller E. E. Hu Y. O. Lebrun L. A. Roume H. Lundin D. (2014). Systematic design of 18S rRNA gene primers for determining eukaryotic diversity in microbial consortia. *PLoS One* 9:e95567. 10.1371/journal.pone.0095567 24755918 PMC3995771

[B15] Karasartova D. Gureser A. S. Ruh E. Turegun-Atasoy B. Calgin M. K. Tasci L. (2018). An alternative DNA extraction method for detection of *Blastocystis* spp. in human fecal samples. *Exp. Parasitol.* 186 36–41. 10.1016/j.exppara.2018.01.019 29438666

[B16] Ma Z. Wang R. Wang M. Zhang X. Mao H. Tan Z. (2018). Short communication: variability in fermentation end-products and methanogen communities in different rumen sites of dairy cows. *J. Dairy Sci.* 101, 5153–5158. 10.3168/jds.2017-14096 29779558

[B17] McAllister T. A. Bae H. D. Jones G. A. Cheng K. J. (1994). Microbial attachment and feed digestion in the rumen. *J. Anim. Sci.* 72 3004–3018. 10.2527/1994.72113004x 7730196

[B18] Mcsweeney C. Mackie R. (2012). *Commission on Genetic Resources for Food and Agriculture. Micro-organisms and Ruminant Digestion: State of Knowledge, Trends and Future Prospects.* Rome: FAO.

[B19] Pollock J. Glendinning L. Wisedchanwet T. Watson M. (2018). The madness of microbiome: attempting to find consensus “best practice” for 16S microbiome studies. *Appl. Environ. Microbiol.* 84:e02627-17. 10.1128/AEM.02627-17 29427429 PMC5861821

[B20] Quast C. Pruesse E. Yilmaz P. Gerken J. Schweer T. Yarza P. (2013). The SILVA ribosomal RNA gene database project: improved data processing and web-based tools. *Nucleic Acids Res.* 41 D590–D596. 10.1093/nar/gks1219 23193283 PMC3531112

[B21] Rantakokko-Jalava K. Jalava J. (2002). Optimal DNA isolation method for detection of bacteria in clinical specimens by broad-range PCR. *J. Clin. Microbiol.* 40 4211–4217. 10.1128/jcm.40.11.4211-4217.2002 12409400 PMC139681

[B22] Rognes T. Flouri T. Nichols B. Quince C. Mahe F. (2016). VSEARCH: a versatile open source tool for metagenomics. *PeerJ* 4:e2584. 10.7717/peerj.2584 27781170 PMC5075697

[B23] Rosewarne C. P. Pope P. B. Denman S. E. McSweeney C. S. O’Cuiv P. Morrison M. (2011). High-yield and phylogenetically robust methods of DNA recovery for analysis of microbial biofilms adherent to plant biomass in the herbivore gut. *Microb. Ecol.* 61 448–454. 10.1007/s00248-010-9745-z 20838785

[B24] Salonen A. Nikkila J. Jalanka-Tuovinen J. Immonen O. Rajilic-Stojanovic M. Kekkonen R. A. (2010). Comparative analysis of fecal DNA extraction methods with phylogenetic microarray: effective recovery of bacterial and archaeal DNA using mechanical cell lysis. *J. Microbiol. Methods* 81 127–134. 10.1016/j.mimet.2010.02.007 20171997

[B25] Schloss P. D. Westcott S. L. Ryabin T. Hall J. R. Hartmann M. Hollister E. B. (2009). Introducing mothur: open-source, platform-independent, community-supported software for describing and comparing microbial communities. *Appl. Environ. Microbiol.* 75 7537–7541. 10.1128/AEM.01541-09 19801464 PMC2786419

[B26] Vaidya J. D. van den Bogert B. Edwards J. E. Boekhorst J. van Gastelen S. Saccenti E. (2018). The effect of DNA extraction methods on observed microbial communities from fibrous and liquid rumen fractions of dairy cows. *Front. Microbiol.* 9:92. 10.3389/fmicb.2018.00092 29445366 PMC5797766

[B27] Walters W. Hyde E. R. Berg-Lyons D. Ackermann G. Humphrey G. (2015). Improved bacterial 16S rRNA gene (V4 and V4-5) and fungal internal transcribed spacer marker gene primers for microbial community surveys. *mSystems* 1 e9–e15. 10.1128/mSystems.00009-15 27822518 PMC5069754

[B28] Whitehouse N. L. Olson V. M. Schwab C. G. Chesbro W. R. Cunningham K. D. Lykos T. (1994). Improved techniques for dissociating particle-associated mixed ruminal microorganisms from ruminal digesta solids. *J. Anim. Sci.* 72 1335–1343. 10.2527/1994.7251335x 8056682

[B29] Yu Z. Morrison M. (2004). Improved extraction of PCR-quality community DNA from digesta and fecal samples. *BioTechniques* 36(5) 808. 10.2144/04365ST04 15152600

